# Measurement of glomerular filtration rate by dynamic contrast‐enhanced magnetic resonance imaging using a subject‐specific two‐compartment model

**DOI:** 10.14814/phy2.12755

**Published:** 2016-04-13

**Authors:** Aaryani Tipirneni‐Sajja, Ralf B. Loeffler, Niels Oesingmann, John Bissler, Ruitian Song, Beth McCarville, Deborah P. Jones, Melissa Hudson, Sheri L. Spunt, Claudia M. Hillenbrand

**Affiliations:** ^1^ Department of Diagnostic ImagingSt. Jude Children's Research HospitalMemphisTennessee; ^2^ Department of Biomedical EngineeringUniversity of MemphisMemphisTennessee; ^3^ Siemens Medical SolutionsNew YorkNew York; ^4^ Division of NephrologySt. Jude Children's Research HospitalMemphisTennessee; ^5^ Department of Pediatric NephrologyLe Bonheur Children's HospitalMemphisTennessee; ^6^ Department of PediatricsVanderbilt University School of MedicineNashvilleTennessee; ^7^ Department of OncologySt. Jude Children's Research HospitalMemphisTennessee; ^8^ Department of PediatricsStanford University School of MedicineStanfordCalifornia

**Keywords:** Glomerular filtration rate, kidney, MRI, tracer, Wilms' tumor

## Abstract

Measuring glomerular filtration rate (GFR) by dynamic contrast‐enhanced (DCE) magnetic resonance imaging (MRI) as part of standard of care clinical MRI exams (e.g., in pediatric solid tumor patients) has the potential to reduce diagnostic burden. However, enthusiasm for this relatively new GFR test may be curbed by the limited amount of cross‐calibration studies with reference GFR techniques and the vast variety of MR tracer model algorithms causing confusion on the choice of model. To advance MRI‐based GFR quantification via improved GFR modeling and comparison with associated ^99m^Tc‐DTPA‐GFR, 29 long‐term Wilms' tumor survivors (19.0–43.3 years, [median 32.0 ± 6.0 years]) treated with nephrectomy, nonnephrotoxic chemotherapy ± radiotherapy underwent MRI with Gd‐DTPA administration and a ^99m^Tc‐DTPA GFR test. For DCE‐MRI‐based GFR estimation, a subject‐specific two‐compartment (SS‐2C) model was developed that uses individual hematocrit values, automatically defines subject‐specific uptake intervals, and fits tracer‐uptake curves by incorporating these measures. The association between reference ^99m^Tc‐DTPA GFR and MR‐GFRs obtained by SS‐2C, three published 2C uptake, and inflow–outflow models was investigated via linear regression analysis. Uptake intervals varied from 64 sec to 141 sec [96 sec ± 21 sec] and hematocrit values ranged from 30% to 49% [41% ± 4%]; these parameters can therefore not be assumed as constants in 2C modeling. Our MR‐GFR estimates using the SS‐2C model showed accordingly the highest correlation with ^99m^Tc‐DTPA‐GFRs (*R* ^2^ = 0.76, *P* < 0.001) compared with other models (R^2^‐range: 0.36–0.66). In conclusion, SS‐2C modeling of DCE‐MRI data improved the association between GFR obtained by ^99m^Tc‐DTPA and Gd‐DTPA DCE‐MRI to such a degree that this approach could turn into a viable, diagnostic GFR assay without radiation exposure to the patient.

## Introduction

Cure of many cancers depends on proper dosing of antineoplastic chemotherapy. Many chemotherapeutics as well as their metabolites undergo renal clearance. A precise measurement of renal function is critical in order to administer an optimal dose to achieve the desired effect and limit toxicity. The clearance component of renal function is described by the glomerular filtration rate (GFR) (Smith 1951; K/DOQI clinical practice guidelines for chronic kidney disease, 2002). Traditionally, GFR has been approximated using formulas based on serum and urine creatinine or measured using cystatin C or a radiotracer (Lameire et al. 2006; Nehus et al. 2013). Creatinine has significant limitations in part because it depends on muscle mass and is secreted by the kidney. While cystatin C is useful, it also can be inaccurate in some forms of cancer and treatment (Oc et al. 2014). Radiotracers likewise have limitations in routine care including delays related to test scheduling, performance, and interpretation as well as radiation exposure.

For solid tumor patients, routine anatomical imaging using magnetic resonance imaging (MRI) has become a standard tool in clinical medicine. Efforts to combine anatomical information with renal functional data include dynamic contrast‐enhanced (DCE) MRI (Prasad 2006) that uses gadolinium (Gd) chelates to measure split renal function without exposing patients to ionizing radiation. Using imaging data to estimate renal function is complex because of the unique structural and functional features of the kidney. Several models based on tracer intrarenal kinetics have been proposed for estimating GFR by DCE‐MRI (Hackstein et al. 2003; Annet et al. 2004; Buckley et al. 2006; Lee et al. 2007; Sourbron et al. 2008; Zhang et al. 2008; Bokacheva et al. 2009; Tofts et al. 2012). Most of the published models consist of various modifications of a two‐compartment (2C) model, consisting of renal vascular and tubular compartments (Hackstein et al. 2003; Annet et al. 2004; Buckley et al. 2006; Sourbron et al. 2008; Tofts et al. 2012).

Simpler 2C models, or uptake models, describe only the inflow of contrast from the renal vascular space into the tubules (Hackstein et al. 2003, 2005; Tofts et al. 2012). These studies implemented a 90 sec fixed uptake interval in volunteers (Hackstein et al. 2005; Tofts et al. 2012), and a 110 sec fixed interval in a patient cohort (Hackstein et al. 2003). The duration of the uptake interval directly impacts GFR quantification (i.e., an underestimation can lead to an overestimation of GFR), therefore accuracy of these uptake models depends heavily on determining the correct uptake interval. Furthermore, a Gd‐DTPA pharmacokinetic study has shown that uptake intervals vary significantly among patients, depending on the degree of renal insufficiency (Krestin 1992). These findings suggest that models that use a fixed uptake interval for all subjects might not be appropriate for MR‐based GFR quantification, especially in patients with impaired renal function. In order to avoid selecting an uptake interval, more recent 2C models account for both, tubular inflow and outflow of tracer (Annet et al. 2004; Sourbron et al. 2008). However, inflow–outflow models may produce inaccurate results due to insufficient acquisition duration and inadequate temporal resolution (Michaely et al. 2008).

Reference GFR methods measure plasma renal clearance. For calculating MR‐based GFR, the tracer plasma concentration has to be determined from the whole‐blood tracer concentration and the hematocrit (Hct) value. Most MR‐GFR studies assumed a fixed Hct obtained from literature (Sourbron et al. 2008; Tofts et al. 2012), while only a few measured the actual subject's Hct (Hackstein et al. 2005). Incorporating the subject's Hct is very important as even small deviations in Hct could produce large errors in MR‐GFR quantification (Tofts et al. 2012).

To resolve these issues, we propose a simple 2C uptake model, called subject‐specific (SS‐2C) model that utilizes a subject‐specific uptake interval and Hct for quantifying MR‐GFR. The objective of this study was to identify the DCE‐MRI model that demonstrates the highest association with reference GFR measurements obtained by ^99m^Tc‐DTPA serum clearance. For this purpose, the MR‐GFR values estimated with the proposed model and other published 2C uptake and inflow–outflow models were correlated with ^99m^Tc‐DTPA reference measurements in a cohort of long‐term survivors of unilateral Wilms' tumor.

## Materials and Methods

### Subjects

This imaging study is part of an institutional pilot study that investigated the prevalence of renal dysfunction in 46 adult, long‐term (>10 years) survivors of unilateral Wilms' tumor who had undergone nephrectomy during their cancer treatment and were over 18 years of age at study entry. Study enrollment exclusion criteria included surgery on the remaining kidney, tumor recurrence during or after the first cancer treatment regimen, exposure to nephrotoxic chemotherapy, radiotherapy to the lungs or structures other than the kidney that had been removed, known genetic renal disease, renal impairment due to structural or functional abnormalities, pregnancy, and contraindications to gadolinium‐enhanced MRI imaging. The study protocol was approved by the institutional review board (IRB), and informed written consent was obtained from all study participants. The participants consisted of two subgroups which received different therapy for Wilms' tumor: (1) irradiated – nephrectomy, chemotherapy, and whole abdominal radiotherapy, and (2) non‐irradiated – nephrectomy, chemotherapy, and no radiotherapy.

### Hct measurements

A blood sample was drawn from all subjects. A complete blood count (CBC) was obtained by using a Beckman Coulter hematology analyzer. Hct results were calculated from the red blood cell count (RBC) and mean cell volume (MCV) according to the following equation: Hct (%) = (RBC x MCV)/10.

### Reference GFR measurements

GFR values were estimated by the ^99m^Tc‐DTPA serum clearance method (Rodman et al. 1993). ^99m^Tc‐DTPA was administered at 3 mCi per 1.73 m^2^ body surface area, followed by a 10 mL saline flush through a peripheral arm vein. Blood samples were drawn at 5, 10, 15, and 30 min and 1, 2, 3, 4, and 6 h after injection. Radioactivity in serum samples was determined by a gamma counter. GFR estimates using the ^99m^Tc‐DTPA serum clearance method were calculated by using a compartmental model with a multiexponential function (Rodman et al. 1993). The reference measurements were done with a median period of ‐1 ± 7 days relative to the MRI examinations.

### MRI measurements

All MRI measurements were performed on a 1.5T Avanto MRI scanner (Siemens Medical Solutions, Erlangen, Germany). Noncontrast multibreath‐hold T1‐weighted 2D FLASH imaging was performed along the long axis of the kidney with the following parameters: TE = 4.22 msec, TR = 90 msec, flip angle = 60°, matrix = 256 × 256, voxel size = 1.56 × 1.56 × 3.00 mm^3^, and 24–30 slices with no slice gap. This sequence covered the entire kidney and was used to measure renal volumes required for calculating the mean kidney GFR.

For the DCE acquisition, a series of T1‐weighted 2D saturation‐recovery‐FLASH oblique coronal image sets were acquired using the following parameters: TE = 0.98 msec, TR = 347 msec, TI = 177 msec, flip angle = 8°, matrix = 192 × 192, voxel size = 2.6 × 2.1 × 8.0 mm^3^, and with 20% slice gap. Each image set consisted of one slice through the aorta and four slices covering the kidney. For contrast administration, a bolus of 0.05 mmol/kg body weight Gd‐DTPA (Magnevist^®^; Bayer HealthCare Pharmaceuticals, Wayne, NJ) was injected intravenously, followed by a 20 mL saline flush at a rate of 4 mL/sec. A total of 135 time points, 10 before contrast injection (precontrast) and the remaining after contrast injection (postcontrast) were obtained. The temporal resolution was 1.7 sec and the acquisition time was 17 sec for precontrast images and 217 sec for postcontrast images. Precontrast measurements served as baseline scans to obtain an accurate estimate of contrast concentration in tissue. Precontrast images were acquired under breath‐hold, whereas postcontrast images were acquired with subjects holding their breath as long as possible, followed by shallow breathing.

The study subjects underwent DCE‐MRI only when their reference GFR measurements were more than 60 mL/min per 1.73 m^2^ as determined by either serum creatinine or ^99m^Tc‐DTPA (Rodman et al. 1993; Levey et al. 1999). This criterion was chosen and approved by the IRB because the Gd injection was not clinically indicated and hence the risk of Gd potentially causing renal problems or nephrogenic systemic fibrosis in people with severely impaired kidneys was deemed too high (Grobner 2006). However, recent literature showed that Gd‐based agents are clinically safe in patients with severely reduced renal function (GFR: 15–29 mL/min per 1.73 m^2^) when low contrast doses are applied (Chrysochou et al. 2009).

### MRI data analysis

After the MR examination, all images were analyzed on a computer workstation. For GFR estimation, all precontrast and postcontrast DCE‐MRI images were registered, regions of interest (ROIs) were drawn, and the mean signal intensity in each region was recorded by using a custom‐developed signal analysis tool (Oesingmann and Goldman 2007). The aortic signal was sampled by drawing a rectangular ROI in the suprarenal abdominal aorta, and the renal signal was obtained by drawing an ROI covering the whole kidney parenchyma. As the contrast concentration and signal intensity change are linearly related at low concentrations (Jones et al. 2005), Gd concentrations in the aorta and kidney were represented by signal changes between the corresponding precontrast and postcontrast images.

A normal renal signal intensity–time curve after Gd injection can be characterized by three phases (Grenier et al. 2008): (1) a first peak, which corresponds to the “vascular‐to‐glomerular first‐pass,” wherein the contrast agent enters the vascular space of the cortex; (2) a slowly ascending segment, which ends at a second peak and corresponds to the uptake phase or filtration phase (wherein the contrast agent flows from the glomerulus into the tubule); and (3) a slowly descending segment, called the “outflow phase,” during which the contrast agent leaves the kidney. The end of the second peak is considered the end‐of‐uptake point. In this study, the uptake point was automatically determined on a subject‐by‐subject basis as follows:

Gadolinium concentrations in the aorta were divided by (1 – Hct) to obtain plasma concentrations (Tofts et al. 1999). An arterial input function (AIF) was generated by fitting a two‐gamma variate function to the aortic signal curve (Davenport 1983), which represents the first pass and the recirculation of the contrast agent. To automatically determine the subject‐specific end‐of‐uptake point, the signal curve in the kidney parenchyma was first smoothed (Duan et al. 2011) and the peak of the upslope curve was detected using the signal processing toolbox in Matlab (Mathworks, Natick, MA). If the renal signal curve shows a very well‐defined uptake phase and outflow phase, the program picks the end of the upslope curve directly before the start of the outflow phase, that is, before a significant amount of contrast leaves the kidney parenchyma. If the renal curve shows a plateau or a very slowly increasing curve between the initial upslope curve and the excretory phase, the script picks the end of the initial upslope curve as the end‐of‐uptake point, because the plateau indicates that a significant amount of contrast is leaving the parenchyma. An experienced operator inspected the series of dynamic images for all cases and verified that the program picked the time point where the following features were observed: cortex and medulla were completely filled, that is, no corticomedullary differentiation and the contrast starts excreting from the kidney by accumulating in the collecting ducts.

After the automated selection of the end‐of‐uptake points, the kidney curves were modeled by using a 2C uptake model that takes into account the tracer arterial delay and bolus dispersion in the glomeruli (Annet et al. 2004), and fits the data from the postaortic rise (Hackstein et al. 2003), which is the measurement before the first increase in the aortic signal after the contrast injection, only up to the respective uptake phases. MR‐GFR values were also calculated by using the 2C model with various fixed uptake intervals reported in literature: 90 sec and 110 sec postaortic rise (Hackstein et al. 2003; Tofts et al. 2012), and using a 2C inflow–outflow model (Sourbron et al. 2008). GFR values were calculated using subject‐specific Hct values and a fixed Hct value of 41% from literature (Tofts et al. 2012), to demonstrate the effect of the Hct value on GFR quantification.

All model fits were performed using the Levenberg–Marquardt nonlinear least squares algorithm in Matlab to minimize the residual difference between the model constructed and the measured signal curves. The equations for the 2C uptake and inflow–outflow models can be found in Method S1. All kidney models calculate GFR values per volume of renal parenchyma (GFR_V_ in min^−1^), and these values were converted into mean MR‐GFR values (in ml/min) by multiplying with their respective renal parenchymal volumes. The volumes were calculated by segmenting the renal parenchyma (excluding the renal pelvis) from the noncontrast multislice T1‐weighted images using Amira (Visage Imaging, Inc., San Diego, CA).

Statistical analysis was performed using the statistical toolbox in Matlab. The mean ± standard deviation (SD) and range of reference GFR values and MR‐GFR values obtained by using various 2C uptake and inflow–outflow models with fixed and subject‐specific Hct values were reported. Linear regression and Bland–Altman analysis were used to quantitatively evaluate differences between ^99m^Tc‐DTPA reference GFRs and MR‐GFRs obtained by using various uptake and inflow–outflow models.

## Results

MRI examinations were performed in 39 of the 46 study subjects (five of them declined to participate and two were unable to fit in the scanner). No contrast was injected in four of the 39 subjects: two subjects had a reference GFR of less than 60 mL/min per 1.73 m^2^, one subject had asthma, and one subject had a high creatinine level. The DCE‐MRI data was not usable in five subjects: one subject had polycystic kidney disease and contrast injection was unsuccessful in four subjects. One subject declined to undergo the ^99m^Tc reference GFR measurement. Thus, our study for quantifying GFR and comparing with the reference measurements consisted of 29 subjects [18 female and 11 male; median age, 32.0 ± 6.0 (range: 19–43.3) years]. The median age at treatment was 3.0 ± 2.5 years (range, 0.1–12.3 years), and median time since the end of therapy was 29.6 ± 5.3 years (range, 15.5–36.3 years). Of the 29 subjects, 15 received radiation at the time of treatment (eight female and seven male; median age, 34.0 ± 6.2 years) and 14 did not (10 female and four male; median age, 29.5 ± 4.7 years). Hct values of all subjects ranged from 30% to 49% (mean, 41% ± 4%) and measured renal parenchymal volumes ranged from 158 to 333 mL (mean, 241 ± 52 mL).

**Figure 1:**
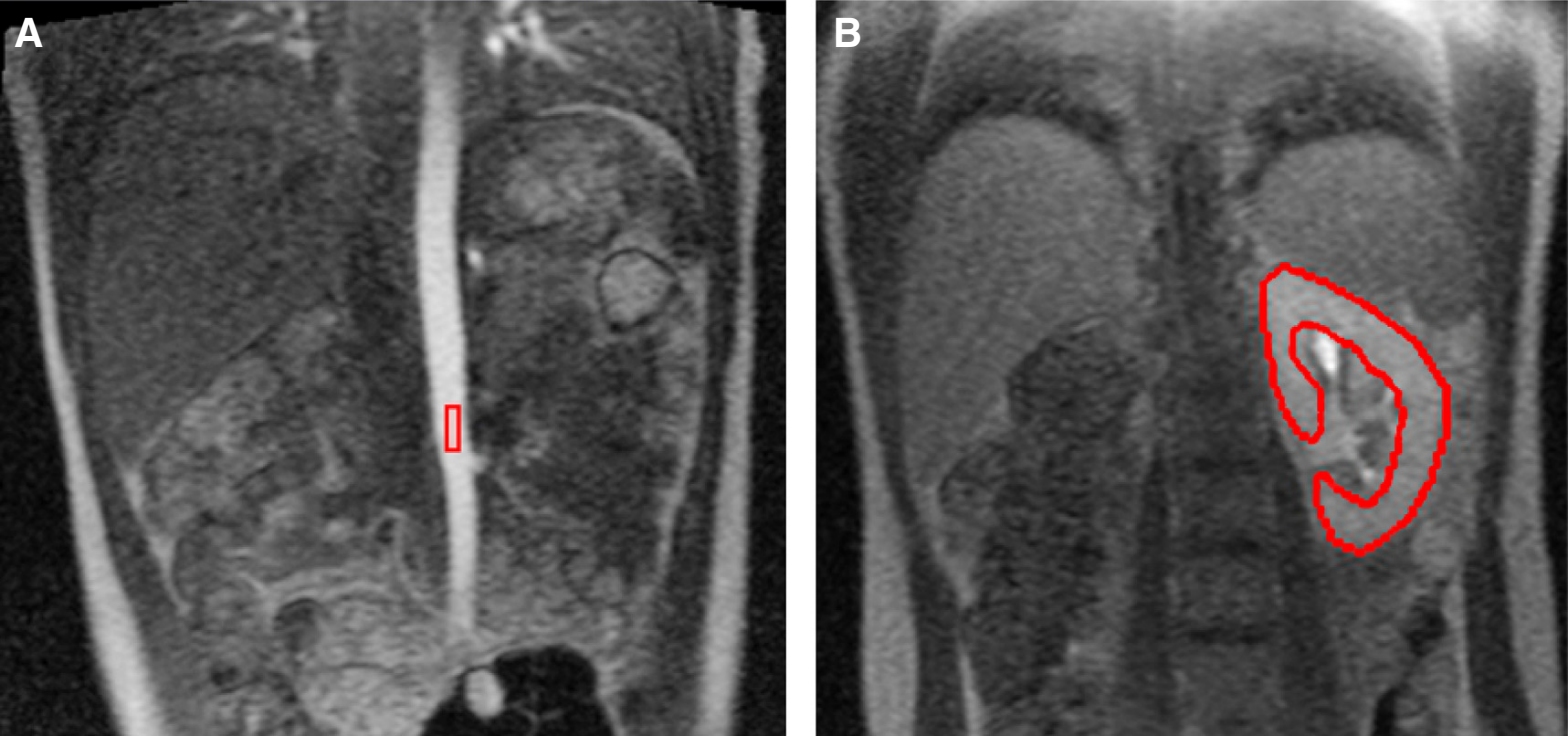
Regions of interest drawn for glomerular filtration rate (GFR) quantification. (A) and (B) show contours (red) drawn for obtaining signal intensity curves in the aorta and renal parenchyma, respectively.

**Figure 2:**
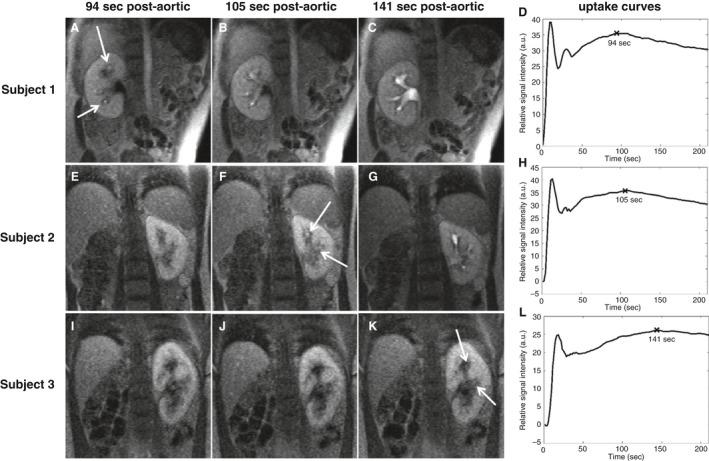
Dynamic contrast‐enhanced MR images and renal uptake curves in three study subjects. Examples shown represent a normal (Subject 1, uptake interval 94 sec), a slower (Subject 2, 105 sec), and very slow (Subject 3, 141 sec) uptake phase. Each row shows parenchymal images for the subject at time points 94 sec, 105 sec, and 141 sec postaortic, and the corresponding uptake curve. The white arrows point to the areas of early excretion of contrast agent from the renal parenchyma into the collecting ducts. The “X” mark on the uptake curves (D, H, L) represent the end‐of‐uptake points (i.e., the maximum parenchymal enhancement) corresponding to images A, F, K, respectively.

Figure 1 shows the ROIs drawn in the abdominal aorta and the whole renal parenchyma for obtaining signal intensity curves to quantify GFR. Figure 2 shows DCE‐MRI images of three subjects at three time points postcontrast injection and their respective uptake curves. The uptake curve of subject 1 (Fig. 2D) is representative for the contrast uptake timing in the parenchyma of a healthy person. At 94 sec postaortic, subject 1 demonstrates no corticomedullary differentiation and has tracer excretion in the collecting ducts and calyces (Fig. 2A). In contrast, subject 2 (Fig. 2E) shows vanishing corticomedullary differentiation and subject 3 (Fig. 2I) shows prominent corticomedullary differentiation at 94 sec. Furthermore, subject 2 demonstrates tracer excretion in ducts and calyces at 105 sec (Fig. 2F) and subject 3 shows very late tracer excretion at 141 sec postaortic (Fig. 2K). Among the 29 subjects, the uptake interval varied from 64 sec to 141 sec with a mean interval of 96 ± 21 sec postaortic.

**Figure 3:**
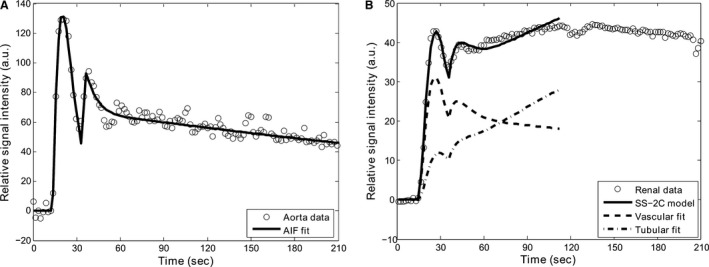
(A) Arterial input function fit and (B) 2C model fit with subject‐specific uptake interval and Hct value (SS‐2C) in a study subject. Also shown are renal vascular and tubular fits as calculated by the model equations (Method S1). Note that the fit curves are drawn only up to the end‐of‐uptake point because the model is valid only up to this point.

**Figure 4:**
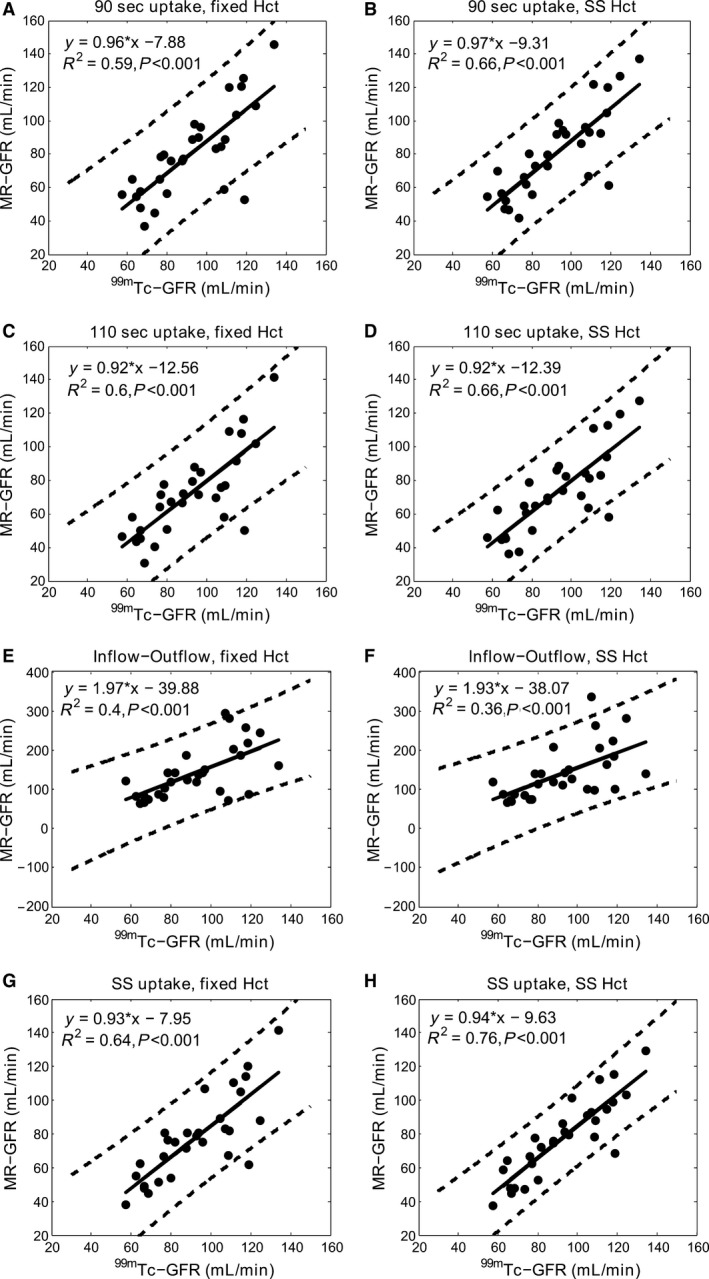
Linear regression plots between ^99m^Tc‐ glomerular filtration rate (GFR) (reference) and MR‐GFR values (in mL/min) for 90 sec fixed uptake model (A, B), 110 sec fixed uptake model (C, D), inflow–outflow model (E, F), and subject‐specific (SS) uptake model (G, H). Plots in the first column (A, C, E, G) are using fixed Hct at 41% and in the second column (B, D, F, H) are using SS Hct for all MR‐GFR models. Solid lines denote regression lines and dashed lines show 95% confidence intervals (± 2SD) for the data. Results of the linear regression analysis performed between ^99m^Tc‐GFR and MR‐GFR values are also shown for all models.

**Figure 5:**
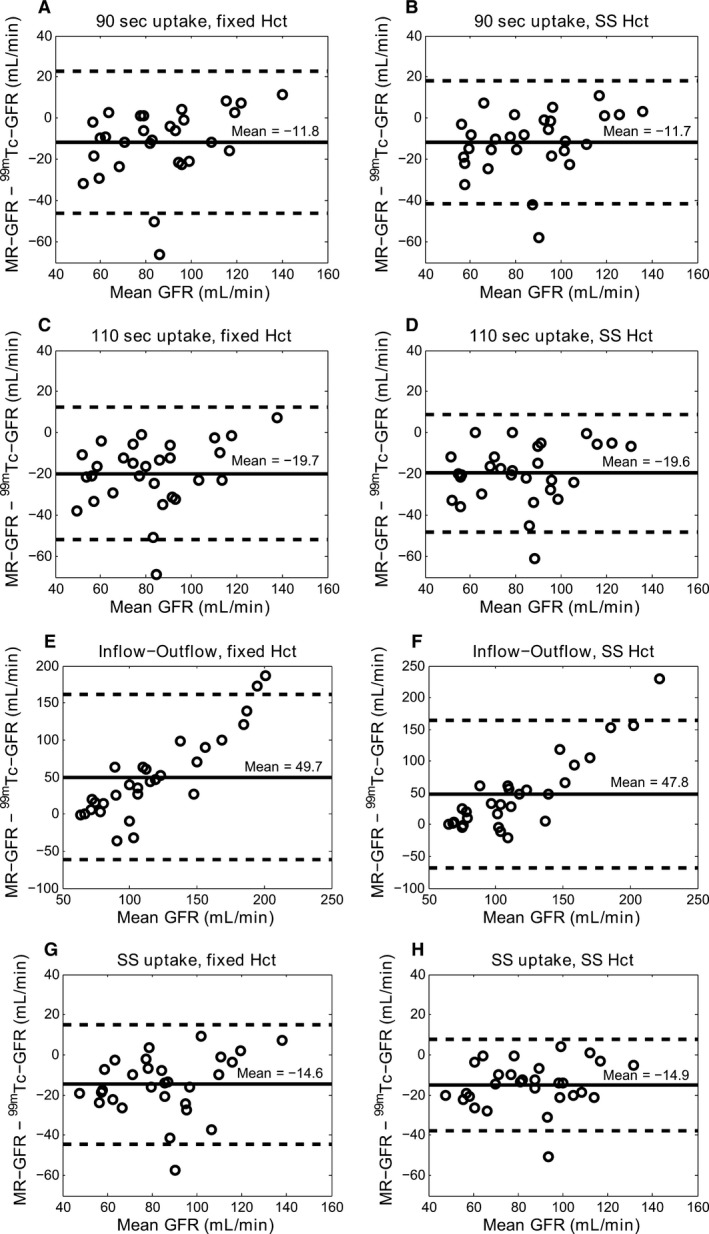
Bland–Altman plots between ^99m^Tc‐ glomerular filtration rate (GFR) (reference) and MR‐GFR values (in ml/min) for 90 sec fixed uptake model (A, B), 110 sec fixed uptake model (C, D), inflow–outflow model (E, F), and subject‐specific (SS) uptake model (G, H).Plots in the first column (A, C, E, G) are using fixed Hct at 41% and in the second column (B, D, F, H) are using SS Hct for all MR‐GFR models. Solid lines show the mean bias and dashed lines show 95% confidence intervals (±2SD) for the data. Of note, all plots are not equally scaled.

All AIF fits, 2C uptake, and inflow–outflow models fits produced *R* ^2^ > 0.95. Figure 3 shows an AIF fit with the two‐gamma variate function and the 2C kidney fit, using the SS‐2C uptake model for a representative subject. Table 1 shows the mean, SD, and range of the ^99m^Tc‐DTPA‐based reference GFR measurements, and MR‐GFR values calculated using various 2C uptake and inflow–outflow models with fixed and subject‐specific Hct values. Figure 4 shows linear regression analysis between various MR‐GFR models and the ^99m^Tc‐DTPA reference GFR method. All uptake models produced a slope close to one and the correlation coefficients ranged from 0.59 to 0.76 when compared with the ^99m^Tc‐DTPA reference measurements. The inflow–outflow model greatly overestimated GFR values and showed a very poor correlation when compared with the reference values. The plots also show the impact of using fixed and subject‐specific Hct values on the GFR quantification. Incorporating subject Hct values greatly improved the correlation (R^2^) for all uptake models. Among all MR models, GFR estimates obtained by the SS‐2C model that utilizes subject‐specific uptake intervals and Hct values produced the highest correlation with the reference GFR values (*R* ^2^ = 0.76, *P* < 0.001). Bland–Altman comparison between reference GFR values and MR‐GFR values estimated using various models are shown in Figure 5. All uptake models underestimated GFR values compared to the reference method with a mean bias ranging from −19.7 to −11.7 mL/min. The SS‐2C model underestimated the GFR with a mean bias of ‐14.9 mL/min and demonstrated tighter confidence intervals compared with other MR‐models.

**Table 1:** Range and mean ± SD of GFR measurements obtained with the ^99m^Tc reference method and different MR‐based GFR models

Method	Range (mL/min)	Mean ± SD (mL/min)
^99m^Tc‐GFR	57–134	92 ± 21
*MR‐GFR with fixed Hct:*		
SS	38–141	78 ± 25
90 sec	37–146	81 ± 27
110 sec	31–141	73 ± 25
Inflow–outflow	63–294	142 ± 67
*MR‐GFR with SS Hct:*		
SS	37–129	77 ± 23
90 sec	41–137	81 ± 26
110 sec	36–128	73 ± 24
Inflow‐outflow	65–336	140 ± 69

^99m^Tc‐GFR, reference GFR; MR‐GFR, GFR measured by MRI with fixed and subject‐specific (SS) parameters (Hct, uptake intervals); SD, standard deviation.

## Discussion

MRI is an emerging modality for studying renal anatomy and function in a single examination. Recent literature documents various advances in determining renal functional parameters such as diffusion and perfusion using MRI (Zhang et al. 2014). However, for GFR estimation, research is still ongoing to find a reliable, easy to obtain, and clinically applicable model for accurate quantification. The presented SS‐2C model improved the correlation of MR‐based GFR quantification with the reference method, and hence demonstrates that MRI has the potential to provide a comprehensive and noninvasive alternative to conventional renal function tests.

Our study describes a simple 2C uptake model to quantify GFR values using DCE‐MRI in a cohort of long‐term survivors of unilateral Wilms' tumor. The study demonstrates that the contrast uptake duration in the kidneys can vary greatly among subjects. The GFR results using our proposed SS‐2C model yielded the best correlation with reference GFR values suggesting that accounting for subject‐specific uptake times is important. Incorporating subject‐specific uptake times into the model was straightforward and added no additional processing time because we implemented an algorithm that automatically determined the intervals using common functions in Matlab.

Our results also demonstrate that using subject‐specific Hct greatly improves correlation with reference GFR values. All uptake models showed improved correlation when normal Hct values from the literature were replaced with the true Hct measured individually for each subject. Error propagation analysis shows that a 1% deviation from the true Hct value induces a 0.72% error in estimating GFR (Tofts et al. 2012). Although our subjects were all considered healthy (GFR >60 mL/min per 1.73 m^2^), their Hct values varied from 30% to 49% (corresponding to a maximum of 37% deviation from the literature Hct value of 41%). Hence, it is very important to measure true Hct values before the MR‐GFR scan in order to calculate correct GFR values, especially in patient cohorts who may exhibit an even wider Hct range. To the best of our knowledge, there is no previous study in the literature that quantified GFR values using fixed Hct and actual subject's Hct and compared them with reference GFR measurements.

The regression analysis between GFR values from all uptake models and ^99m^Tc‐GFR produced a slope close to, but smaller than 1. On the other hand, the MR‐GFR values estimated using the inflow–outflow model were greatly overestimated as observed in some previous studies (Buckley et al. 2006; Tofts et al. 2009), and showed a very poor correlation with the reference measurements. Although the inflow–outflow model removes the ambiguity of selecting the uptake interval, it imposes constraints to the acquisition: a temporal resolution of 5 sec or less, and an acquisition time of 220 sec or more to be able to accurately quantify GFR with an error of less than 10% in healthy volunteers (Michaely et al. 2008). In our study, temporal resolution was sufficient with 1.7 sec and acquisition time was 217 sec, which is close to the suggested 220 sec. The observed large overestimation of GFR values in our study group may be due to the fact that the optimal acquisition time depends on physiology, that is, plasma and tubular mean transit times (Michaely et al. 2008). Due to the wide variation in uptake intervals in our study cohort, we hypothesize that much longer acquisition times than 220 sec may be required when using a 2C inflow–outflow model to accurately quantify GFR. However, longer acquisition times may not be clinically feasible.

The end‐of‐uptake points picked by the automated script were verified by an experienced operator via visual assessment. There was agreement between automated uptake point selection and visual inspection in 90% of the cases (26 out of 29). Disagreement in three cases (Figure S2) was attributed to respiratory motion induced signal intensity changes, misalignment of the ROI with the kidney parenchyma, or poor signal‐to‐noise ratio. Using the end‐of‐uptake time points provided by the reader for these three cases, did not improve the correlation. These observed issues could be potentially avoided by performing accelerated 3D imaging for a higher signal‐to‐noise ratio (SNR) in combination with radial sampling for motion compensation (Wright et al. 2014), and employing advanced coregistration and segmentation approaches. Unfortunately, these advanced imaging techniques were not available to us at the time this study was initiated. However, we believe that the model fit algorithms described in this study are equally applicable to 2D or 3D acquisitions, and hence can be applied to 3D imaging as long as there is sufficient SNR and temporal resolution.

GFR values obtained by using the SS‐2C uptake model appeared lower than those estimated using the ^99m^Tc‐DTPA serum clearance method. This underestimation might be due to inflowing blood into the imaging slice of the aorta that artificially enhances the signal in the aorta leading to overestimation of the AIF and eventually underestimation of GFR (Peeters et al. 2004). Another possible reason for this difference is that the serum clearance of ^99m^Tc‐DTPA may overestimate GFR values by ~30% compared with urinary clearance because of leakage of tracer into the extravascular space as discussed in a report by Klassen et al. (Klassen et al. 1992). Moreover, the method used for ^99m^Tc‐GFR calculations also demonstrated overestimation of actual clearance if there is limited sampling schedule (Rodman et al. 1993).

A major advantage of our study design was that all subjects had previously undergone a unilateral nephrectomy. With only one kidney remaining, all model‐derived MR‐GFR values could be directly correlated with the ^99m^Tc‐DTPA measurements without having to account for errors based on estimating the split renal function for the reference method. Hence, our study is likely more accurate than previous studies in establishing and evaluating the DCE models and validating the correlation with the ^99m^Tc‐GFR method.

It should be noted, that all MR‐models used in this study are capable of estimating renal perfusion (Method S1) which may also contribute to MR‐based functional renal assessment and further guide therapy. MR‐based renal perfusion values were not reported here as our study design did not include a reference method for comparison.

There are some limitations to this study. First, per institutional policy, only study subjects with a reference GFR of more than 60 mL/min per 1.73 m^2^ were eligible for contrast injection. This limited our study to an essentially healthy subject cohort, and we were therefore not able to test our model in individuals with critically impaired renal function. Second, we used a 2D imaging sequence that acquired only four slices of the kidney and reported the mean GFR estimated from these four thick slices as the whole kidney GFR. However, there is a recent publication showing that single slice and whole kidney analysis produced comparable GFR results (Winter et al. 2014). Nevertheless, inadequate coverage may not lead to accurate GFR estimates in cases of severe chronic kidney disease that display heterogeneous renal lesions. These issues could potentially be overcome by using above‐mentioned modern 3D imaging techniques that provide thin slice coverage of the whole kidney. However, traditional 3D imaging available at the initiation of this study reduced the temporal resolution that is crucial in capturing the dynamics of the tracer's uptake, and was therefore not chosen. Third, some of our DCE‐MRI images were very noisy. High SNR is essential for obtaining high‐quality signal curves (i.e., smooth and low‐jitter, thereby avoiding ambiguity in the selection of the end‐of‐uptake point) and eventually accurate GFR values. Fourth, the ^99m^Tc‐DTPA serum measurements were obtained at a median period of −1 ± 7 days relative to the MRI examinations; therefore, there may be physiologically induced variations in MR‐ and ^99m^Tc GFR values which may impact the correlation. Finally, the cohort size was relatively small.

In conclusion, this study demonstrates that DCE‐MRI using the proposed SS‐2C model improved the correlation of the MR‐based GFR estimation with the reference method compared with previously reported MR‐GFR models. As DCE‐MRI‐based GFR values correlate well with those from ^99m^Tc‐DTPA scans, DCE‐MRI could develop into an alternative, diagnostic GFR test especially for patients already undergoing MRI imaging of the abdomen. Benefits may accrue by eliminating the additional time and cost associated with separate GFR quantification, but of even greater benefit, especially for pediatric patients, is that this method does not expose the subject to ionizing radiation. The DCE‐MRI acquisition takes less than 5 min; it can be easily integrated into a standard MRI exam and may replace post injection waiting periods thereby requiring no extra time at all.

## Conflict of Interest

None declared.

## Supporting information




**Figure S1.** Illustration of the 2C kidney model. Ao_p_, tracer concentration in the aortic plasma; P and T, tracer concentrations in renal plasma and tubular compartments, respectively; RPF, renal plasma flow; GFR, glomerular filtration rate. The dashed line indicates tubular outflow for the inflow–outflow model.
**Figure S2**. Dynamic contrast‐enhanced MR images and renal uptake curves in the three study subjects in whom the automated uptake interval selection (A, D, and G) and the visual assessment (B, E, and H) are in disagreement. The black and red “X” marks on the uptake curves (C, F, and I) represent the end‐of‐uptake points picked by the automated script and by an experienced operator, respectively. The operator selected the earliest time point that shows no corticomedullary differentiation and tracer excretion in the collecting ducts (solid white arrows). However, the time points picked by the automated script already showed some tracer in the collecting ducts (dashed white arrows); this means that a noticeable amount of contrast agent left the parenchyma even though the medulla has not completely enhanced yet.
**Methods S1.** Derivation of tracer kinetic modeling equations.
