# Supplementary material for: Measurement of glomerular filtration rate by dynamic contrast‐enhanced magnetic resonance imaging using a subject‐specific two‐compartment model
Source: Physiol Rep. 2016 Apr 13;4(7):e12755. doi: 10.14814/phy2.12755 (PMC4831325; doi:10.14814/phy2.12755)
Supplement: Supplementary file 1 — Figure S1. Illustration of the 2C kidney model. Aop, tracer concentration in the aortic plasma; P and T, tracer concentrations in renal plasma and tubular compartments, respectively; RPF, renal plasma flow; GFR, glomerular filtration rate. The dashed line indicates tubular outflow for the inflow–outflow model. Figure S2. Dynamic contrast‐enhanced MR images and renal uptake curves in the three study subjects in whom the automated uptake interval selection (A, D, and G) and the visual assessment (B, E, and H) are in disagreement. The black and red “X” marks on the uptake curves (C, F, and I) represent the end‐of‐uptake points picked by the automated script and by an experienced operator, respectively. The operator selected the earliest time point that shows no corticomedullary differentiation and tracer excretion in the collecting ducts (solid white arrows). However, the time points picked by the automated script already showed some tracer in the collecting ducts (dashed white arrows); this means that a noticeable amount of contrast agent left the parenchyma even though the medulla has not completely enhanced yet. Methods S1. Derivation of tracer kinetic modeling equations. [file PHY2-4-e12755-s001.docx]

# Supporting information


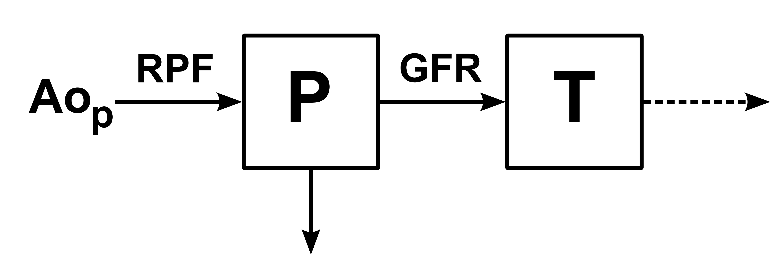


**Figure S1.** Illustration of the 2C kidney model. Ao_p_, tracer concentration in the aortic plasma; P and T, tracer concentrations in renal plasma and tubular compartments, respectively; RPF, renal plasma flow; GFR, glomerular filtration rate. The dashed line indicates tubular outflow for the inflow-outflow model.

# Method S1: Derivation of tracer kinetic modeling equations

The tracer concentration in renal tissue *K(t)* is expressed as a combination of renal plasma *P(t)* and tubular concentrations *T(t)* ([Hackstein et al. 2003](#_ENREF_2)) as shown in Figure S1:

$\boldsymbol{K}\left( \boldsymbol{t} \right)= \boldsymbol{V}_{\boldsymbol{p}}\boldsymbol{P}\left( \boldsymbol{t} \right)+\boldsymbol{T}(\boldsymbol{t})$, (1)

where *V_p_* is the renal plasma volume (ml/100 ml). The plasma concentrations in the aorta *Ao_p_(t)* is obtained from the whole-blood concentration *Ao(t)* and the hematocrit *Hct*:
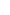

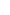


$\boldsymbol{A}\boldsymbol{o}_{\boldsymbol{p}}\left( \boldsymbol{t} \right)\boldsymbol{=Ao(t)/(1-Hct)}$. (2)

The tracer concentration versus time curves of the renal plasma and the aortic plasma are assumed to be the same. However, the curve is shifted and dispersed to consider the transit delay of the contrast from the aorta to the kidney and bolus dispersion in the glomeruli ([Annet et al. 2004](#_ENREF_1)). Hence, the concentration in renal plasma space is expressed as

$\boldsymbol{P}\left( \boldsymbol{t} \right)= \frac{\mathbf{1}}{\boldsymbol{T}_{\boldsymbol{p}}}\int_{\mathbf{0}}^{\boldsymbol{t}} \boldsymbol{A}\boldsymbol{o}_{\boldsymbol{p}}(\boldsymbol{u}-\boldsymbol{\tau})\boldsymbol{e}^{-\frac{(\boldsymbol{t}-\boldsymbol{u})}{\boldsymbol{T}_{\boldsymbol{p}}}}\boldsymbol{du}$, (3)

where *T_p_* is the plasma mean transit time, and
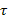
$\tau$ is the tracer transit delay. The amount of tracer filtered into the tubular compartment is assumed to be proportional to the integral of the tracer concentration curve in the renal plasma space as per the definition of clearance ([Hackstein et al. 2003](#_ENREF_2)):

$\boldsymbol{T}\left( \boldsymbol{t} \right)= \boldsymbol{GFR}_{\boldsymbol{V}}\int_{\boldsymbol{0}}^{\boldsymbol{t}} \boldsymbol{P}\left( \boldsymbol{u} \right)\boldsymbol{du}$, (4)

where *GFR_V_* represents the tracer clearance from the plasma space into the nephron, which, in other words, is the GFR per unit volume of tissue. This assumption is true for the uptake models which assume that no contrast leaves the kidney during the analysis. The inflow-outflow model considers tracer outflow from the tubules by incorporating a parameter *T_t_* called tubular mean transit time. Now the tubular concentration is given by:

$\boldsymbol{T}\left( \boldsymbol{t} \right)= \boldsymbol{GFR}_{\boldsymbol{V}}\int_{\mathbf{0}}^{\boldsymbol{t}} \boldsymbol{P}\left( \boldsymbol{u} \right) \boldsymbol{e}^{-(\boldsymbol{t}-\boldsymbol{u})/\boldsymbol{T}_{\boldsymbol{t}}}\boldsymbol{du}.$ (5)

For both uptake and inflow-outflow models, the input parameters are *Hct*, *Ao(t)*, and *K(t)* and output parameters are *V_a_*, *GFR_V_*, $\tau$, and *T_p_* for uptake models and an additional parameter *T_t_*, for the inflow-outflow model. Another functional parameter, RPF or renal perfusion can also be extracted from all models using the following equation:$RPF \left( {{ml}/{100 ml}}/{min} \right)= {V_{p}}/{T_{p}}$.


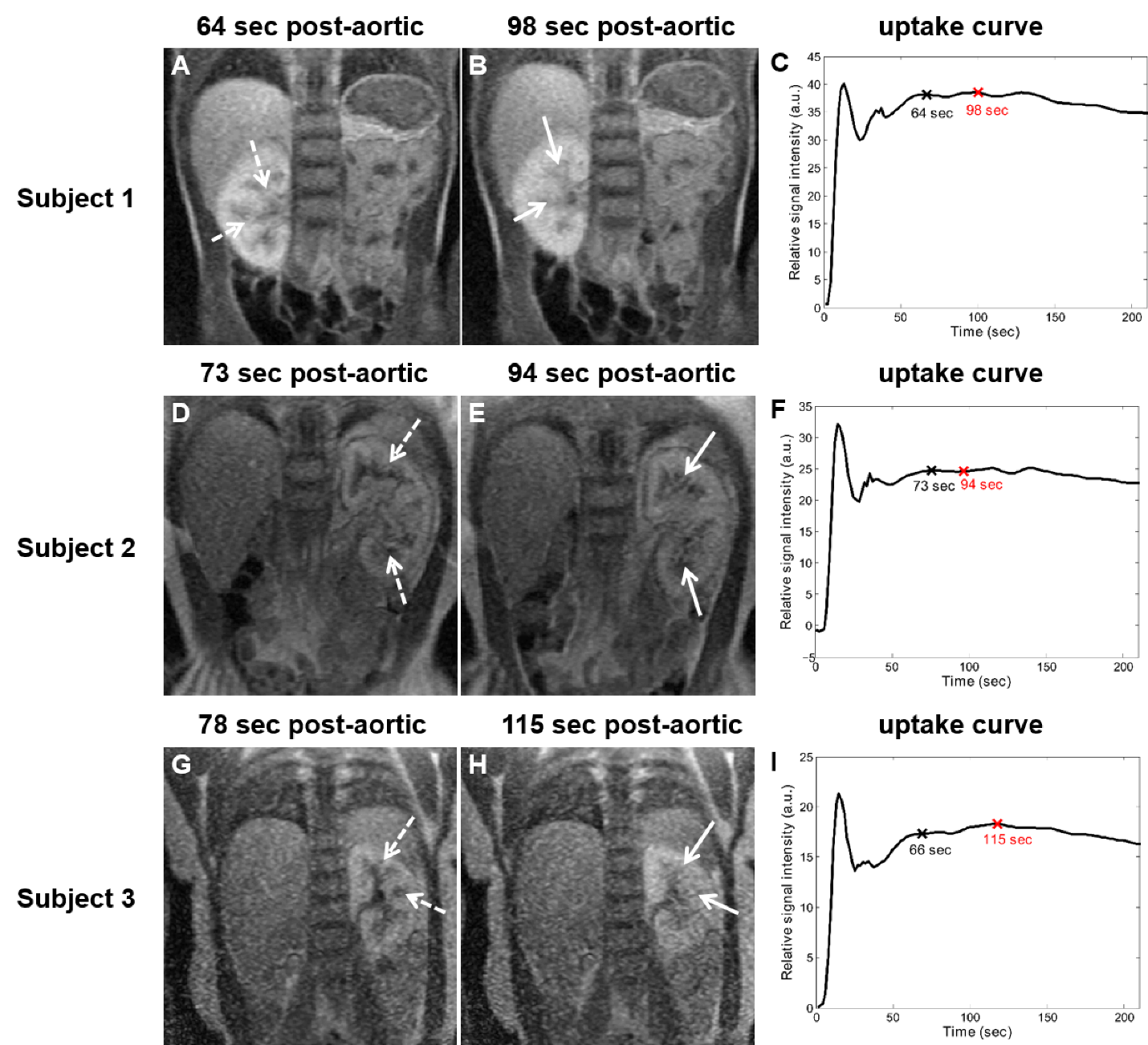


**Figure S2.** Dynamic contrast-enhanced MR images and renal uptake curves in the 3 study subjects in whom the automated uptake interval selection (A, D, and G) and the visual assessment (B, E, and H) are in disagreement. The black and red ‘X’ marks on the uptake curves (C, F, and I) represent the end-of-uptake points picked by the automated script and by an experienced operator, respectively. The operator selected the earliest time point that shows no cortico-medullary differentiation and tracer excretion in the collecting ducts (solid white arrows). However, the time points picked by the automated script already showed some tracer in the collecting ducts (dashed white arrows); this means that a noticeable amount of contrast agent left the parenchyma even though the medulla has not completely enhanced yet.

# References

Annet, L., L. Hermoye, F. Peeters, F. Jamar, J. P. Dehoux and B. E. Van Beers (2004). "Glomerular filtration rate: assessment with dynamic contrast-enhanced MRI and a cortical-compartment model in the rabbit kidney." J Magn Reson Imaging **20**(5): 843-849.

Hackstein, N., J. Heckrodt and W. S. Rau (2003). "Measurement of single-kidney glomerular filtration rate using a contrast-enhanced dynamic gradient-echo sequence and the Rutland-Patlak plot technique." J Magn Reson Imaging **18**(6): 714-725.
